# The Law of the Similars

**Published:** 1885-10

**Authors:** Ad. Lippe

**Affiliations:** Phila.


### THE LAW OF THE SIMILARS.

AD. LIPPE, M. D., PHILA.

In the second number of the first volume of the *Organon*, a quarterly recently published at Liverpool (April, 1878), will be found a paper on this subject; it was the endeavor of that paper to show that the Law of the Similars is a Law of Nature; that it was practically applied by the father of medicine, "Hippocrates;" that Hahnemann was the first physician, who showed that all and every cure ever made was owing to the accidental application of this law, proving the correctness of his assertions by numerous quotations; that it was left to the genius of Hahnemann to establish by a strictly inductive method the only law by which therapeutics can be governed—"*similia similibus curantur.*" There were not wanting men who, from the first introduction of the healing art, attempted a variety of progressive departures from the teachings and practice of the founder of our school; various motives, various shortcomings on their part, caused these departures—but, as they increased their success in curing diseased conditions decreased also, and hence led to still further departures. Time showed very plainly that the chasm between the progressive homœopathicians and the progressive defenders of further departures widened year by year. A late departure was a rejection, or rather an attempt to reject, our accepted formula. The late *British Journal of Homœopathy* claims to have found that the formula "*similia similibus curan-*

*tur*" was not correct, and while that journal for over forty years had, in acceptance of it, displayed this formula on its title-page, it was now proclaimed to be erroneous, and should not read *curantur* but *curentur*. The then journal ceased to exist.

It was hoped that no further departures would be attempted, but these were vain hopes. A final departure from all formulas, a departure from our only law by which therapeutics can be governed, was taken at the last session of the American Institute. We are now informed that the President of said Institute, not satisfied with proposing three subjects for INVESTIGATION, which had been investigated and decided long ago, declared at the banquet of the American Institute at St. Louis that, "*We are a free people, bound by no law.*" As homœopathicians we are governed in our therapeutics by the natural Law of the Similars. If we openly defy this governing law, we cease to be homœopaths, we sink into the ranks of the eclectics. Hippocrates even teaches in the book *De Morbo Sacro* (Epilepsy), "Diseases are generally cured by the very thing that caused them;" a further explanation of this axiom is given in the book *De lucis in homine*, where he says, "Similars cause and cure diseases." "That which causes strangury, cough, diarrhœa, and vomiting is also able to cure these evils." And now, in the year 1885, the President of the American Institute tells us "We are a free people, bound by no law;" and, as silence is always supposed to give consent, it seems as if the President uttered the sentiments of the company he was in. If such a sentiment was uttered at a banquet of "Communists," it would certainly express the sentiments of the men who boast of supporting the red flag; this sentiment would be in keeping with these law-defying members of society at large, and every good and law-abiding member of the community would declare his abhorrence of such a sentiment. And if we are professedly homœopaths, it behooves us to be bound by the law we professedly adopted as the only law by which therapeutics can be governed. The eclectics are to be compared with the lawless Communists—they are, in fact, professionally medical Communists, bound by no law. Even the common school is governed and bound to follow the ever-varying new hypotheses of their school—now it is the germ theory, by and by it will be a new hypothesis to be followed. On the 16th of September, 1885, the French Academy of Medicine discussed the report of Drs. Chantemesse and Rummo on the analysis made by them on Dr. Ferran's cholera vaccine matter. The conclusion arrived at in the report, which was adopted by the Academy, is that the so-called vaccine matter cannot afford protection against cholera.

The declaration of the President of the American Institute, who is also Dean of a Homœopathic (?) College, will not for one moment deter the faithful homœopathicians, who accepted the only law of cure by which therapeutics can be governed, of following up their determination to further develop the means by which this unerring natural law can be applied for the cure of the sick.

How came these increasing and progressive departures from the methods and teachings of Hahnemann to invade and be advocated by the American Institute? How came this disgrace to be enacted, and have the true healers a remedy to preserve them from the taunts of the common school of medicine, on account of such conduct of the President of the American Institute, and by the silence of the assembled members of the Institute itself? The heroic band of true men who in 1844 formed the American Institute of Homœopathy did not, could not, anticipate that in 1885 the Institute by them founded to develop our Healing Art should be so disgraced as it has been. The observing members of this Institute are well aware of two facts—*first,* that members of our school who followed and developed Hahnemann's methods rarely, nay, exceptionally only, did seek admission to the Institute, that for more than a decade the addresses by the Presidents of the Institute as well as the transactions of that body unmistakably showed signs of great progressive departures from the aims the founders of the Institute had in view in 1844; *second,* the large accession of new members of the Institute of late years came from the late graduates of the so-called homœopathic colleges, and would not have aided to disgrace the Institute had they received good, sound homœopathic instructions, to which they were looking forward to and were entitled to receive at professedly homœopathic colleges, chartered for the purpose of teaching Homœopathy by a generous and liberal people. What were they taught? Certainly not Homœopathy, but a sort of eclecticism, which would be of no use to the young graduate, as under the application of what he was taught he would fail to cure, and, having been deceived by his teachers, having been made to believe that he was taught Homœopathy, a minority of educated men would begin the study of Homœopathy *de novo* and become useful members of the profession but not members of the Institute, a majority of uneducated graduates would blindly condemn Homœopathy pure, and, merely adopting the name for show, fall into the same vile practice of eclecticism as was taught and practiced by their professors, and they joined the Institute of course. The root of the evil which presents itself to us is that the professedly homœopathic colleges do not teach Homœopathy, that the majority of the graduates of these schools swell the well-organized ranks of the Institute to the detriment of our Healing Art. Are these colleges chartered by the people for the purpose of teaching Homœopathy "bound by no law," as the President of the Institute expresses himself, or are they not amenable to the law of the land? Are they not bound under their charter, to teach in harmony with the Law of the Similars? That is the question which presents itself to the homœopathic profession. In remorse we leave the question not only to the homœopathic profession but to the people at large and especially to the trustees of the professedly homœopathic colleges who represent the people and who are expected to see to it that the provisions of the charter are fulfilled faithfully, that the Law of the Similars and the methods of Hahnemann are taught in their school, excluding the pernicious teaching that we are bound by no law, and see to it that the graduates are prepared to serve the community as true healers always governed by the Law of the Similars.

